# Approaches to Address the Anemia Challenge

**DOI:** 10.1016/j.tjnut.2023.07.017

**Published:** 2023-09-14

**Authors:** Cornelia U. Loechl, Ananya Datta-Mitra, Lindy Fenlason, Ralph Green, Laura Hackl, Laura Itzkowitz, Marion Koso-Thomas, Denish Moorthy, Victor Ochieng Owino, Helena Pachón, Nicole Stoffel, Michael B. Zimmerman, Daniel J. Raiten

**Affiliations:** 1 Division of Human Health, International Atomic Energy Agency, Vienna, Austria; 2 Department of Pathology and Laboratory Medicine, University of California, Davis, Davis, CA, United States; 3 Bureau for Global Health, USAID, Washington, DC, United States; 4 USAID Advancing Nutrition, John Snow Inc., Arlington, VA, United States; 5 Eunice Kennedy Shriver National Institute of Child Health and Development, National Institutes of Health, Bethesda, MD, Unites States; 6 Food Fortification Initiative, Emory University, Atlanta, GA, United States; 7 Laboratory of Human Nutrition, Department of Health Sciences and Technology, ETH Zurich, Zu¨rich, Switzerland; 8 MRC Human Immunology Unit, MRC Weatherall Institute of Molecular Medicine, University of Oxford, John Radcliffe Hospital, Oxford, UK

**Keywords:** anemia, interventions, food-based, nutrition-specific, nonnutritional

## Abstract

Anemia is a multifactorial condition; approaches to address it must recognize that the causal factors represent an ecology consisting of internal (biology, genetics, and health) and external (social/behavioral/demographic and physical) environments. In this paper, we present an approach for selecting interventions, followed by a description of key issues related to the multiple available interventions for prevention and reduction of anemia. We address interventions for anemia using the following 2 main categories: *1*) those that address nutrients alone, and, *2*) those that address nonnutritional causes of anemia. The emphasis will be on interventions of public health relevance, but we also consider the clinical context. We also focus on interventions at different stages of the life course, with a particular focus on women of reproductive age and preschool-age children, and present evidence on various factors to consider when selecting an intervention—inflammation, genetic mutations, nutrient delivery, bioavailability, and safety. Each section on an intervention domain concludes with a brief discussion of key research areas.

## Introduction

Anemia, defined as low hemoglobin (Hb) concentration in the blood below a specified cut-off point adjusted for age, sex, physiologic status, and altitude above sea level [1], remains a critical global public health problem. As described by other papers in this supplement, we need to consider anemia as a condition that develops within an ecology [2] (intertwining biology and mechanistic aspects of nutrients [3] with both the health status and underlying factors—physical, economic, social, behavioral, demographic, and environmental). The causes of anemia are multifactorial, and the decision-making framework presented by Williams et al. [4] considers how to systematically determine the etiology of anemia in populations. We herein address interventions for anemia using the following 2 main categories: *1*) those that address nonnutritional causes of anemia, and *2*) those that address nutrients alone. The emphasis will be on interventions of public health relevance, but we also consider the clinical context.

### Selection of interventions

The complexity of anemia requires multisectoral collaborative efforts to understand and sustainably reduce it [[2], [3], [4]]. After a comprehensive assessment of anemia and its causes (as detailed earlier in the supplement by Williams et al. [4]), the next step is the selection of a single or multiple interventions for anemia. We select a single or a package of appropriate efficacious and effective interventions that are implemented within the context of individual and population factors (socioeconomic, political, and cultural influences, and physical environment and climate). The feedback on how interventions are being implemented and their impact on health and nutrition status is informed by activities related to monitoring, evaluation, and research. In our paper, we present evidence of effectiveness behind various nonnutritional and nutritional interventions for anemia. Within the evidence, we highlight elements of the intervention that are relevant to its implementation, including its design, targeting strategy, the geographic and population groups that are targeted, and social and behavioral factors. The analytic process outlined within this approach lies within the epidemiologic domain; it needs to be accompanied by actions with respect to operational (logistics and resources for implementation) and sociopolitical (social, political, cultural, and organizational) factors [5]. The research gaps highlighted in the various sections are derived from discussion between various members of the Anemia Task Force, a deliberative body of anemia experts [2], who comprise the coauthors of this manuscript and the 3 other papers in this supplement [[2], [3], [4]]. The studies included in the review were derived from knowledge of the Anemia Task Force members, and the conclusions represent their expert opinion. We did not conduct a systematic literature search. The following sections provide overviews of available approaches to interventions for both nondietary and nutrition-specific anemia.

## Nondietary interventions for prevention and treatment of anemia

Nondietary or nonnutritional interventions for the treatment of anemia include a broad range of approaches to apply to an individual or at the population level as a single clinical intervention or broader public health programs. In this section, we address nondietary interventions for the treatment of anemia among disorders with the highest global burden of disease including malaria, helminthiasis, tuberculosis (TB), HIV, acute blood loss, and hemolytic genetic disorders.

### Malaria preventive chemoprophylaxis

Malaria is a parasitic infestation responsible for close to half a million deaths a year [6]. Regions in the world with higher transmission rates have an increased risk of anemia in the entire population with the greatest impact in infants and young children [7,8]. In lower transmission settings, symptomatic malaria and resulting anemia may occur at all ages, although preschool-age children and pregnant women are more likely to have anemia. At all levels of transmission, malaria (all Plasmodium species) is an important contributor to maternal anemia during pregnancy and poor birth outcomes [[9], [10], [11]]. The reciprocal relationships between iron intake/physiology and malaria (both in the parasite and the host) are well recognized [3]. Malaria during pregnancy poses a unique challenge for prevention and control of anemia because of the significant impact on both the mother and the fetus [12]. We address anemia caused by malaria infection with intermittent preventive regimens of chemoprophylaxis with drugs, such as intermittent preventive treatment with sulfadoxine-pyrimethamine (IPTp-SP) [11]. However, where the sulfamethoxazole-pyrimethamine combination is the main therapeutic drug in use, folic acid may lead to increased incidence and lower parasite clearance. In addition, the WHO guidelines integrate the use of long-lasting insecticide nets as a prevention tool with effective diagnosis and management of cases in a holistic approach to malaria management in pregnant women and those of child-bearing age [12].

In areas where *Plasmodium falciparum* malaria transmission is stable and pregnant women are semi-immune, infections are mostly asymptomatic, making diagnosis a challenge and hampering targeted prevention programs through the use of malaria screening. Current evidence supports chemoprophylaxis with pyrimethamine/dapsone in both primigravid and multigravida women to reduce parasitemia thereby increasing Hb [9,11,12]. When implemented effectively, IPTp-SP reduces maternal malaria episodes, maternal and fetal anemia, placental parasitemia, low-birth weight, and neonatal mortality [9,11,12]. In the face of emerging drug resistance and questions around the safety of repeated doses of IPTp-SP during pregnancy, alternate regimens for malaria control in pregnancy include the use of rapid diagnostic screening during the antenatal period, with immediate treatment if the mother tests positive. Evidence that a newer therapy, such as artemisinin-based combination therapy with dihydroartemisinin-piperaquine is superior to the current standard of 2 or 3 doses of IPTp-SP is minimal [9]. Like in pregnant women, practitioners can prevent anemia in infants and young children by controlling malaria with the use of vector control, deployment of insecticide-treated bed nets, prompt and accurate diagnosis of illness, and appropriate use of effective antimalarial drugs [13]. Social and behavior change factors that impact malaria control include norms around net usage, fear of side effects from chemoprophylaxis, and lack of adherence because of poor understanding of the difference between prevention and treatment [14]. Depending on the type of intervention and the context, we will need to consider and address different priority factors, including the timing, safety, and efficacy of interventions to improve iron status [[15], [16], [17]].

However, one reason that ecologic approaches are important is that in individuals with glucose-6-phosphate dehydrogenase (G6PD) deficiency, primaquine causes dose-dependent hemolysis [18]. Without widespread screening for the presence of G6PD deficiency, use of low dose primaquine in endemic regions to reduce population-level transmissibility poses a risk among individuals not previously diagnosed [19]. With G6PD deficiency, hemolysis can cause severe anemia, fatigue, jaundice, and hemoglobinuria. There is no universal consensus for recommendations, although some countries have instituted a once-weekly regimen, which has a relatively safer impact on G6PD deficiency variants with less severe hemolytic disease. As with the other genetic disorders, future directions for treatment include genetic molecular considerations to correct the defect with a small molecule activator, decreasing the red blood cells’ (RBCs) susceptibility to hemolysis under oxidative stress [20].

### Antihelminth treatment

As covered by Brittenham et al. [3], intestinal worm infestations affect >2 billion people worldwide [21]. The common vernacular for intestinal worms refers to “helminths,” which represents a diverse group of parasitic species including roundworms (*Ascaris lumbricoides*), whipworms (*Trichuris trichiura*), and hookworms (*Necator americanus* and *Ancylostoma duodenale*). Other intestinal infestations, such as amebiasis (e.g. *entamoeba histolytica*) and other parasites, such as *giardia* are associated with intestinal inflammation and blood loss, which can lead to anemia, especially in high-risk population subgroups of young children and pregnant women [22,23]. Clinicians generally refer to these species collectively, as we diagnose and treat them similarly. The species of the parasite, maternal nutritional status, and presence of comorbid conditions, such as malaria and HIV, influence the extent of morbidity [24,25]. For instance, 38% of women in the Wondo Genet district in Southern Ethiopia with intestinal parasitic infections were found to have substantially higher prevalence of anemia than noninfected women (55.6% compared with 15.4%, *P* < 0.001) [26]. Population-based deworming programs recommended by WHO consider mass drug administration, targeted chemotherapy, and selective therapy [27]. Systematic reviews of studies addressing interventions for helminth infestation have shown mixed results. Salam et al. [28] summarized results of 4 trials that measured the impact of a single dose of antihelminthics with third trimester anemia and reported that although all showed a statistically significant impact, the strength of the evidence was low because of attrition bias and inconsistent results between the 4 studies.

Studies in sub-Saharan Africa demonstrated improvements in Hb as a result of deworming in children. This is also seen in studies from India [29]. However, the improvements were context-specific (i.e., based on the severity of their anemia and of the burden of parasitic load). The results of these interventions in different geographic regions raise the importance of context even for “universal” public health interventions (e.g., mass deworming programs with or without nutrient supplementation). It is not simply a matter of gender and age group, but also ethnicity [30,31]. A review of the effect of deworming school children shows that these interventions result in a reduction of anemia at the community level, but a multifaceted approach that combines hygiene programs and coadministration of micronutrients including iron and vitamin A is needed [31]. This is exemplified in operational guidance from the Pan-American Health Organizations that highlights the focus on prevention and promotion in interventions for control of helminth infestation: *1*) focusing work on water, sanitation, and household-related factors; *2*) reducing environmental risk factors; *3*) improving health of migrating populations; *4*) reducing inequity because of sociocultural factors and gender; *5*) reducing poverty in endemic populations; and *6*) setting up surveillance and risk assessment systems [32].

### Treatment of anemia of inflammation caused by TB and HIV

The inflammation associated with both TB and HIV impacts iron homeostasis and results in anemia [[33], [34], [35]]. As described by Brittenham et al. [3], anemia of chronic inflammation is mediated through the increase in hepcidin synthesis through a complex mechanism affecting iron homeostasis and erythropoiesis [34,36,37]. High-disease burden and poor prognosis are strongly associated in more severe cases of anemia. Both TB and HIV have increased morbidity and mortality when anemia is present [35].

One key to the choice of treatment is the differentiation of iron-deficiency anemia (IDA) from anemia of inflammation, which is characteristically present in TB and HIV and the iatrogenic anemia associated with treatment of TB. The anemia and inflammatory response caused by TB infection have been shown to persist beyond 60 d even with the initiation of anti-TB treatment [38]. Ezeamama et al.’s [39] exploration of the complexity of the intersection of HIV, the chronic inflammatory response, and treatment suggests that interventions for the anemia of chronic disease improve recovery of patients on highly active antiretroviral therapy including enhanced quality of life. The management of TB and HIV, the diagnosis and treatment approach to the associated anemia must include determination of etiology (e.g., differentiation between nutritional causes including iron deficiency and genetic hemoglobinopathies) to inform context-specific, safe, and efficacious interventions. Novel therapies that target the specific proteins and receptors responsible for the disruption in the erythropoiesis regulation associated with these diseases are not yet commercially available [40].

### Blood transfusions

Women in Asia and sub-Saharan Africa have an increased risk of dying from blood loss in the postpartum period compared with their counterparts in other regions of the world [41,42]. Despite significant strides in postpartum hemorrhage prevention and treatment, the burden of anemia and death during this period remains a global health challenge [42]. A survey of national programs across Asia, Latin America, and Africa showed that although countries have the elements in place to address postpartum hemorrhage, challenges and barriers in policy, programs (availability, accessibility, and affordability), and practice mitigate widespread uptake [43]. However, widespread adoption of comprehensive, evidence-based, up-to-date guidelines would need to effectively occur in the healthcare systems of the most affected populations to have a public health impact. Blood transfusions used to correct the presence of anemia have the potential to be lifesaving, particularly when loss of Hb is acute, as in postpartum hemorrhage and with severe blood loss in trauma, shock, infections such as malaria, and other emergencies. In otherwise healthy pregnant women with mild, moderate, and severe anemia, the use of ferric carboxymaltose infusions have been found to be safe and effective for ≤6-wk post-transfusion [44]. In instances of chronic anemia, a gradual replacement of RBCs through blood transfusions is safer and more physiologically beneficial than intravenous (IV) iron infusions [45]. The definition and parameters for classification of anemia in children (Supplemental Table 1) considers the age of the child, thereby impacting the decision to intervene [46]. Clinicians can use the WHO survey data on anemia thresholds for age groups ranging from infancy to school-age children as a reference to guide treatment [47].

### Delayed cord clamping

Outside of blood loss secondary to maternal acute loss, neonatal and infant anemia is most common because of the hereditary and autoimmune causes and inadequate intake, absorption, and assimilation of iron in infancy and childhood. Immediate cord clamping at birth causes blood loss in newborns by as much as 25–35 mL/kg, leaving them with deficits that reduce iron stores and cause anemia early in life [[48], [49], [50], [51]]. WHO (2014) guidelines [52] recommend delayed umbilical cord clamping (not earlier than 1 min after birth) for improved maternal and infant health outcomes, especially ferritin in infants [48,53].

### Management of inherited RBC disorders

Sickle cell disease and α- and β-thalassemia (along with G6PD deficiency, reviewed along treatment of malaria) [54] comprise a large source of nondietary anemia because of disordered erythropoiesis and/or increased erythrocyte fragility [[55], [56], [57]]. Supportive care such as blood transfusions and pain medication have long been the mainstay of treatment and management of sickle cell anemia and its complications. With the emergence of hydroxyurea, the care, disability, and survival of individuals with the most severe form of this disorder changed dramatically [58]. Cost is the most prohibitive factor limiting the widespread use of hydroxyurea and other drugs with the ability to address the pathophysiology and clinical impact of sickle cell disease. Low-resource regions of the world with a high burden would benefit from the availability of inexpensive therapeutics and a drug pipeline focused on medications with complete or at least partial capability of rescuing more red cells from Hb S polymerization and destruction [59,60].

Transfusion-dependent anemia, including α- and β-thalassemia and other rare anemia, are generally genetic disorders with a low prevalence and a broad range of clinical severity. In many settings, the management of transfusion-dependent anemia is limited to supportive care, including chronic transfusion and iron-chelating therapy. At present, several innovative treatments are in clinical trials for β-thalassemia and other transfusion-dependent anemia [61]. As in the case of sickle cell disease, potential therapeutic interventions with allogeneic stem cell transplantation, drugs, and gene therapy are costly, and have potential toxicity and safety concerns. Rapid progress in the development of drugs to stimulate globin cell production or gene therapy are hopeful considerations [55,62].

As with the other genetic disorders, future directions for treatment include genetic molecular considerations to correct the defect with a small molecule activator, decreasing the RBCs’ susceptibility to hemolysis under oxidative stress [20].

### Other nonnutritional interventions

There exist other nonnutritional interventions that address the underlying determinants of anemia, such as food insecurity and insufficient maternal and child health care services. Because their impact on anemia is indirect, we do not have sufficient direct evidence on their impact on anemia. Nevertheless, these interventions include early childhood development initiatives, child protection, conditional cash transfers, family planning, water, sanitation, and hygiene, and women’s empowerment.

### Summary for nonnutritional interventions

Research has demonstrated the potential benefits of antimalaria treatment, deworming, delayed cord clamping, and therapeutic clinical treatment, such as blood transfusions as interventions to address the issue of anemia to varying degrees, in complex scenarios, and at times with conflicting evidence. In choosing interventions, practitioners should consider and address key factors that inhibit and motivate the desired behaviors. We highlight key questions, research gaps, and recommendations for future directions in the **Supplemental File** (page 3).

## Nutrition-specific interventions

A myriad of factors in the physical and food environment impact on iron status and biology. Although other micronutrients such as folates, vitamin B12, and vitamin A are also associated with anemia, there is a dearth of evidence that parses the impact, on anemia, of public health interventions that deliver these micronutrients, independent of iron status and biology. Hence, we focus on the ecology of iron, although we do present additional evidence on these other micronutrients.

Climate change is increasingly impacting the physical environment’s ability to improve food security, dietary diversity, and diet quality [[63], [64], [65]]. Given the relationship between malaria and iron status, it also illustrates the value of an ecologic approach in making decisions about how best to intervene to improve iron status. Recent evidence has emerged attesting to the direct impact of atmospheric carbon dioxide (CO_2_) on both food quantity (crop yield) and quality (nutritional value of main food crops) [64,66]. Recent estimates show an average of 1% reduction in consumable food calories from 10 major crops including cereals (rice, maize, sorghum, and wheat); legumes (soybean); and oil seeds (oil palm and rapeseed) because of the changing climate and resources [67]. Prolonged CO_2_ accumulation affects nutrient composition in crops, leading to preferential production of soluble sugars and ultimately carbon/nitrogen imbalance [68] and reduced concentration of essential nutrients including protein, calcium, iron, and zinc in major crops [64,[68], [69], [70], [71], [72]]. An estimated 3%–17% of protein, zinc, and iron content are lost in C3 plants in particular (e.g., cassava, soybeans, rice). Although the interactions with nutrient content and bioavailability, as well as health and nutritional status, need deeper investigation, it is clear that climate change will have significant implications on the choices of interventions [73]. The following sections cover factors related to iron intake and status and food-based interventions to address IDA.

### Nutrient–nutrient interactions

The presence of other minerals, especially calcium, in the diet may also influence iron absorption [74] via competition for absorption sites. Research publications suggest that excessive consumption of dairy products, not calcium per se, negatively affects iron absorption [75,76]. Grinder-Pedersen et al. [77] found that calcium from milk and fortified foods had no influence on nonheme iron absorption. A previous study by Hurrell [78] showed that reconstituting roller dried cereal-based complementary foods with milk was associated with reduced iron bioavailability. Zinc, iron, and copper share a common gastrointestinal mechanism for absorption and zinc supplementation can potentially induce iron deficiency [79].

Vitamin A deficiency may cause anemia by impairing iron metabolism. Vitamin A treatment can increase erythropoietin and allow stored iron to be used for erythropoiesis [80]. In vitamin A-deficient populations, improving vitamin A status reduces the prevalence of anemia in most [[80], [81], [82]] but not all [83] studies. The mechanism of the interaction of vitamin A and iron is thought to be mediated through the secretion of the hormone hepcidin, which controls the absorption and mobilization of iron in the body [84]. Increasing vitamin A decreases serum hepcidin, and allows for iron absorption and utilization. The inconsistent results among different studies of vitamin A supplementation could possibly be due to the confounding effect of inflammation from other infectious causes. This highlights the role of ensuring supplementation with other interventions that reduce infection and inflammation, as seen in these studies where controlling for infection and inflammation allows for delivered micronutrients to be absorbed [[85], [86], [87]].

According to Fishman et al. [88], other vitamins, especially folates, vitamin B_12_, riboflavin, vitamin B_6_, and vitamins C and E, are associated with iron status and absorption through various pathways including—

- production of RBCs (vitamins B_6_, B_12_, B_2_, and folate),
- antioxidant role to protect mature RBCs (vitamins C and E), and
- role in intestinal absorption of iron and mobilization of iron stores (vitamins B_2_, A, and C).

### Dietary fiber and phytic acid

Although the role of dietary fiber in mineral absorption has not been conclusively established, recent literature reviews [89,90] suggest that dietary fiber may reduce mineral absorption, including iron, because of binding and physical entrapment [89] and decreased intestinal transit time [90]. Adams et al. [78] noted that the effect of dietary fiber on iron absorption depends on the type of fiber, with insoluble fibers leading to lower iron absorption when compared with whey protein. Phytic acid, which is found in cereals and legumes, is a well-known potent inhibiter of iron absorption and the cause of iron deficiency when diets have a high proportion of cereal and legume content. Dephytinization (removal of phytic acid from food), use of phytases, and competitive blocking with ascorbic acid is used in food-processing techniques to reduce the effect of phytic acid [78].

## Food-based approaches to enhance intake and absorption of iron and other micronutrients

### Adding animal sources of iron or increasing dietary diversity

In many low- and middle-income settings, an over-reliance on plant-based foods limits the quality of diets for the whole population, but specifically for young children, adolescents, and women of reproductive age [68]. Although pulses and cereals are rich in many essential nutrients, they are limited in essential amino acids, certain vitamins (e.g., vitamin B_12_) and bioavailable levels of certain minerals (e.g., zinc and iron). Although only 5%–10% of the nonheme iron predominantly found in plant-based foods is absorbed [81,82], 20%–30% of the heme iron found in animal source foods is absorbed [74,91]. Plant-based diets also contain antinutrient compounds that reduce nutrient absorption [92]; research shows that animal source foods also improve absorption of nonheme iron [93,94]. This suggests the value of improving dietary diversity [80], including animal source foods rich in these nutrients, as part of strategies to improve diet quality.

Nevertheless, results from studies using various types of animal source foods to improve iron intake and status among women and children are mixed. The following are some examples of inconsistency across different animal food sources. Adding small fish and edible spiders to complementary foods in Cambodia had no effect on infant iron status, although the authors note that a subsequent paper that modeled the dietary intakes from the study found that the “intervention foods supplied insufficient iron to meet the iron needs for the infants” [95]. In another study in Kenya, comparing the efficacy of an amaranth-soybean complementary food blend richly fortified with conventional multiple micronutrient premix compared with a blend fortified by adding iron- and zinc-rich edible white termites, the Hb and iron stores of the children consuming the blend with the edible insect were reduced [96]. Another study found that adding fish to dal-based recipes had no effect on iron bioavailability [97]. A study in Vietnam showed that animal source foods, such as pork, chicken, duck eggs, domesticated goose, freshwater shrimp, or tilapia, resulted in improved iron status among women of reproductive age [98].

Pérez-Pérez et al. [99] assessed the effect of a whey-based nutritional supplement on zinc and iron absorption from a plant-based diet in Mexican children. In this randomized crossover study, 16 children consumed either a plant-based diet or the plant-based diet + whey-based nutritional supplement over 2 d. Results showed that the 2 groups did not differ significantly in fractional absorption of iron or total absorbed iron. A systematic review investigating whether a higher consumption of animal flesh foods is associated with better iron status in adults in advanced economies concluded that although the consumption of animal source foods is associated with improved iron status, more evidence is needed on dose–response relationships [100] because some dairy products seem to interfere with iron absorption [75,76,78].

The results of studies improving dietary diversity have also been variable, as exemplified by the following. Recent results from India indicated that dietary diversity score (DDS) had no association with iron status among women [101]. Diana et al. [102] reported that among anemic pregnant women in Indonesia, DDS was positively associated with energy, protein, vitamin A, vitamin C, and zinc adequacy, but it had no significant association with calcium and iron adequacy. A study from Ghana [103] found low DDS among pregnant women, but concluded that preparation for pregnancy and attendance of antenatal care were prerequisites to meeting minimum acceptable diets and that supplementation with iron and folic acid had a greater impact on anemia. In contrast, a systematic review of 16 studies from Ethiopia found that infants who consumed <4 food groups per day were 1.71 times more likely to be anemic [104]. A study from Kenya reported that increased dietary diversity was associated with increased Hb and increased iron intake among pregnant women, but no markers of iron stores were included [105].

Anemia is a complex condition and it is not explained only by a discussion of iron inadequacy and deficiency. The variability in human studies reported is not only because of potential differences in results between women and children and their biological contexts, but also the environmental factors that can affect the design and interpretation of studies.

### Food processing to improve iron bioavailability

As discussed by Brittenham et al. [3], iron bioavailability from food is tightly regulated and differs depending on the chemical form of iron in the food. Most of the studies we present focus on iron; other nutrients that contribute to anemia are not discussed in detail as iron remains the main determinant in the biological processes that lead to anemia. Iron absorption is also subject to both individual iron status and iron bioavailability from the consumed diet; individuals with low-iron stores can absorb iron more efficiently from foods with low-iron bioavailability [106]. Household-level food preparation techniques such as dehulling, peeling, soaking, germination, fermentation, and drying continue to receive research attention because of their potential to improve iron bioavailability. Food safety and quality are thought to improve because of the activation of enzymes such as amylases, phytase, pullulanase, and glycosidases that aid digestion and hydrolysis of antinutrients [107]. Other approaches include addition of exogenous phytase and extrusion cooking.

The results of in vitro studies indicate that the feasibility of implementing these practices is limited. However, there are limited numbers of human studies, and inconsistent results mean these approaches currently are not a focal point of public health programs/interventions. These in vitro studies, outlined in the Supplemental File under the section on “*In Vitro* Studies to Reduce Anti-Nutrients” (pages 5–7) form the evidence base from basic science on the various processes (fermentation and germination, soaking and pressure cooking, dephytinization, abrasive decrotification, and dehulling, use of native or exogenous phytase enzyme, and extrusion cooking) that can be employed to reduce the levels of dietary factors that prevent the absorption of nutrients (i.e., antinutrients). These household interventions have shown results at a smaller scale but they have not been evaluated as a public health intervention.

### Conclusions and future directions for food-based approaches

The biology of nutrition, including iron status, is influenced by many socioeconomic and environmental factors affecting the local and global food system, such as food insecurity and climate change. Although beneficial for meeting requirements for multiple essential micro- and macronutrients, diet quality appears to have mixed results on iron status and bioavailability, potentially because of implementation of these interventions at the household and population level. Evidence regarding the benefit of animal source foods as a stand-alone intervention remains inconclusive, but as they are an important component of programs to increase dietary diversity, their use should be promoted as an integrated package with other dietary interventions. Although enzymes that can break down antinutrient factors hold promise for increasing the bioavailability of iron and other minerals, they will not be widely commercially available in the foreseeable future. One major limitation of food-processing techniques is that most evidence is based on in vitro experiments with no efficacy trials in humans. Although the approaches reviewed have some potential for improving nutrient-responsive anemia, data are limited. To fill the above gaps, consider the following:

• Undertake a dedicated systematic review or technical report to update how cost-effective home-based dietary enhancement strategies impact iron status, and therefore reduce anemia associated with absolute iron deficiency (defined as absent or reduced body iron stores that do not meet the need for iron of an individual but may respond to iron supplementation), in women and children.
• Food matrix (including food structure and dietary fiber) and composition influence iron uptake from foods. Coordinated basic food technology activities would optimize food recipes to maximize iron intake and bioavailability.
• Dietary patterns and food preferences have changed in light of increasing market-based economies even in rural areas. Conduct ethnographic studies to assess acceptability and use of traditional food-processing approaches.
• As a first step, seek more proof of principle animal modeling to determine the efficacy of optimized food recipes on iron bioavailability, with due consideration of the limitations of available animal models.
• Short-lived observational studies with small sample sizes comprise current evidence. We recommend larger longitudinal randomized trials in children and women to determine the efficacy of new food formulations/recipes on iron status and the associated anemia prevalence.

### Food fortification

Fortification is another widely used direct approach to increasing nutrient content, considered cost effective among other micronutrient delivery interventions [108]. Fortification strategies have a long history of success in addressing priority nutrient challenges, such as iodine deficiency disorders [109], neural tube birth defects [110], and vitamin A deficiency-related night blindness [[111], [112], [113], [114]]. We review the experience with food fortification to address nutrient-responsive anemia.

Fortification requires equipment that adds the nutrients to processed food and adequately mixes them together before packaging. In practice, practitioners most often make decisions based on context (i.e., What are staples in the country? Are foods produced or imported at large scale?). These foods include maize flour, milk, oil, rice, salt, and wheat flour [115,116]. The selection of nutrient(s) to add to a fortified food depends on the nutritional needs of the population and the cost of adding the particular nutrient to a particular food. For example, in Argentina, legislation requires the addition of nutrients including iron, which can reduce anemia associated with iron deficiency, and folic acid that can reduce neural tube defects [117]. The number of countries with mandatory fortification legislation ranges from 7 for rice to 129 for salt (Supplemental Table 2). Among these, there is a wide range of countries that includes specific nutrients that contribute to Hb synthesis (i.e., iron, vitamin A, folic acid, vitamin B_12_, and zinc). The levels of nutrients in fortification standards vary widely. The target dose for consumers to receive is a function of the micronutrient content in fortified food and how much the population consumes. Nutrient contents range from 0 to 980 parts per million (ppm) for iron, from 0 to 42 ppm for vitamin A, from 0 to 5.12 ppm for folic acid, from 0 to 0.04 ppm for vitamin B_12_, and from 0 to 102 ppm for zinc. However, nutrients are often combined with other ingredients to stabilize them [118]. The bioavailability of these compounds depends on their inherent properties and how they interact with the diet. Water-soluble iron compounds, such as ferrous sulfate are more bioavailable than compounds that are soluble in dilute acid, such as ferrous fumarate and even less if water insoluble as reduced iron or ferric pyrophosphate [119]. However, the water-soluble iron compounds cause negative sensory changes in the fortification vehicle. Companies and governments select the lowest cost, most technologically compatible iron compounds with an acceptable, but not the highest, bioavailability.

### Mass fortification

Mass fortification requires government regulation and enforcement, and quality control and assurance procedures for the manufacturing industry. An existing production facility, as well as an ongoing production process and supply chain, enable easy and cost-efficient industrial production and trade of the fortified product. The public health context, choice of food vehicle, and program design contribute to successful implementation. The use of innovative delivery platforms, such as the public distribution programs in countries, like India can also reach vulnerable and high-risk populations with fortified products, for example with double fortified salt for anemia [120]. Systematic reviews of efficacy and effectiveness trials suggest that mass fortification with iron improves Hb by 4.2 g/L [121] and 1.9 g/L [110], respectively. The impact of mass fortification on anemia varies accordingly from 41% [121] to 34% [110] in highly controlled studies and country programs, respectively. Mandatory fortification with folic acid in the United States has been shown to eliminate anemia because of folate deficiency [122]. Researchers [123] and government agencies have reviewed the safety of mass fortification, mainly with regards to folic acid, and have largely concurred that the benefits of fortification outweigh any potential risks [[124], [125], [126], [127], [128], [129], [130]].

### Targeted fortification

Targeted fortification refers to the “fortification of foods designed for specific population subgroups” [119]. Two types of products are primarily used for targeted fortification in infants and young children: fortified blended foods (FBFs) and complementary food supplements (CFSs). Typically, practitioners use FBF in humanitarian settings with inadequate or no production capacity to carry out mass fortification and/or a functioning market does not exist to meet the macronutrient and micronutrient needs of the target population. Development settings may also employ FBF to supplement the intake of the population, for example, Incaparina—a fortified blended flour made with corn and soy flours in Guatemala—has reportedly increased iron intake [131]. Wheat–soy blend delivered with micronutrient powders (MNPs) (i.e., home fortification) increased Hb levels by 5.5 g/L and decreased anemia by 54%.

A 2019 review of CFSs focused on one Chinese product (Yingyangbao) found that it increased Hb concentration by 4.4 g/L and reduced anemia prevalence by 45% [132]. Because mixed-fed infants consume the same foods as the rest of the family in many settings, fortified CFS are added to household food intake. These are lipid-based nutrient supplements (LNSs), which include ready-to-use therapeutic foods and ready-to-use supplementary foods. LNS supply energy, protein, essential fatty acids, and micronutrients to meet the needs of individuals [133]. The most appropriate product for targeted fortification depends on the epidemiologic, clinical, and environmental contexts. Evidence shows that fortified CFS improves the micronutrient intakes of infants consuming mixed diets (e.g., breastfed infants aged >6 mo), with positive effects on iron and vitamin A status [134]. Targeted fortification was associated with a 47% reduction in anemia and LNS with a 16% reduced risk of anemia [135]. However, knowledge gaps remain around the recommended nutritional amounts of iron for targeted fortification for children aged <2 y and the most efficacious form of targeted fortification with respect to its impact on anemia.

### Commercial fortification

Commercial, or market-driven, fortification occurs when food manufacturers add one or more micronutrients to a specific food product for a business or brand advantage. These processed products typically reach a smaller portion of the population than those reached by fortified staple foods, and many times, they are not the groups most vulnerable to micronutrient inadequacies and deficiencies. Government regulation ensures that the fortification of these products is safe and will not lead to undesirably high intakes of micronutrients, but it is important to confirm that the marketing of these products responds to public health interests, namely that the consumption of these products will not increase the prevalence of noncommunicable diseases, as these food products tend to be highly processed.

### Biofortification

WHO defines biofortification as “the process by which the nutritional quality of food crops is improved through agronomic practices, conventional plant breeding, or modern biotechnology” [136]. As a result, harvested biofortified crops have higher nutrient levels than nonbiofortified crops. In comparison, the nutritional enhancement of fortified foods occurs postharvest, during the food-processing stage.

Researchers have conducted proof-of-concept development trials with many different crops and nutrients. The nutrient levels in crops were increased through agronomic practices (i.e, iodine in alfalfa [137]); conventional plant breeding (e.g., provitamin A in cauliflower [138]); or modern biotechnology (e.g., thiamine in rice [139]). A subset of crops has been biofortified with one or 2 nutrients through conventional plant breeding for global delivery. The following staple crops have been released in 42 countries: bananas/plantains, beans, cassava, cowpea, lentil, maize, pearl millet, Irish potato, rice, wheat, sorghum, and sweet potato (Table 1) [140]. In addition, 26 countries have National Biofortification Policies and Programs [141].

**TABLE 1:** Biofortified crops released in countries

Crop	Nutrients	Countries where released
Banana/plantain	Vitamin A	2 (Democratic Republic of Congo, Burundi)
Beans	Iron	15 (Panama, Nicaragua, Honduras, Guatemala, El Salvador, Colombia, Brazil, Bolivia, Tanzania, Rwanda, Kenya, Democratic Republic of Congo, Burundi, Uganda, Zimbabwe)
Cassava	Vitamin A	6 (Brazil, Sierra Leone, Ghana, Democratic Republic of Congo, Cameroon, Nigeria)
Cowpea	Iron, zinc	3 (Brazil, Zimbabwe, India)
Lentil	Iron	4 (Syria, Nepal, Bangladesh, India)
Maize	Vitamin A	12 (Brazil, Zambia, Togo, Tanzania, Rwanda, Mali, Ghana, Democratic Republic of Congo, Cameroon, Zimbabwe, Malawi, Nigeria)
Maize	Zinc	6 (Nicaragua, Honduras, Guatemala, El Salvador, Colombia, Bolivia)
Pearl millet	Iron	3 (Togo, Niger, India)
Irish potato	Iron, zinc	0 (No variants released for public use, all variants are still in testing phase)
Rice	Zinc	6 (Nicaragua, El Salvador, Colombia, Indonesia, Bangladesh, India)
Sorghum	Iron, zinc	1 (India)
Sweet potato	Vitamin A	28 (Peru, Panama, Nicaragua, Guatemala, Colombia, Brazil, South Korea, Indonesia, Timor-Leste, Zambia, Tanzania, South Africa, Rwanda, Mozambique, Madagascar, Kenya, Ghana, Ethiopia, Cote d’Ivoire, Burundi, Burkina Faso, Uganda, Angola, Malawi, Bangladesh, China, India, Nigeria)
Wheat	Zinc	7 (Mexico, Brazil, Bolivia, Nepal, Bangladesh, Pakistan, India)

Released biofortified crops have elevated contents of one or multiple nutrients: iron, vitamin A, and/or zinc (Table 1). Plant breeders set targets for the additional amount of nutrients to add to select crops through biofortification [142]. These targets are as follows:

• Forty-four and 30 ppm of added iron for beans and pearl millet, respectively;
• Fifteen and 30 ppm of added provitamin A for cassava, maize, and sweet potato, respectively;
• Twelve ppm of added zinc for rice and wheat.

With biofortification, conventional breeding increases nutrient contents by breeding with cultivars that have naturally high contents of key nutrients. Regular consumption of biofortified foods increases micronutrient intake, nutrition, and health, as is seen with increased iron and zinc with biofortified pearl millet [143], iron in common beans [144], and vitamin A in biofortified orange sweet potatoes [145]. The integration of biofortified crops into agricultural value chains and food systems, alongside those creating consumer desirability for such products, will increase the possibility that biofortified crops are readily available and affordable in local markets throughout the year.

The main challenges to increasing the production and consumption of biofortified crops is time and confirming yield and economic benefit are at least equivalent to that of the traditional crops. The health impacts of biofortification are yet to be proven on a large scale [146], with some measures of positive effect on vitamin A status associated with use of orange-fleshed sweet potato. It takes time to widely introduce biofortified traits into public plant breeding programs, scale up production, and build consumer demand [142]. Given the urgency of climate change and its impact on the micronutrient content of crops, broader consideration of introduction of biofortified crops into policies and programs is needed [147]. That said, 2 key aspects related to introducing biofortified crops must be studied before its introduction—documented health impacts using biomarkers and the potential yield and economic benefits to farmers in low- and middle-income countries, who face increasing fluctuations in yield and its antecedent benefits as a result of climate change. Subsequently, future programmatic work needs to focus on increasing the percentage of total staple food supplies that are biofortified [148]. There is also a need to study the commercial viability of biofortified crops and whether, in a commercial context, farmers can supply high-yield, climate-smart, and nutrient dense varieties to capture a significant market share and improve nutrient status of the population [146]. This meets the goal of providing more nutritious diets, rather than adequate calories [142].

### Key research questions on fortification and biofortification

Although the impact of mass fortification cannot be separated out from other interventions that deliver micronutrients, the effects of mass fortification should be considered through the perspective of providing additional intakes of micronutrients supplied through the fortified foods. When measuring changes in intake (e.g., dietary and household consumption surveys), exposure (e.g., urinary iodine concentration, and serum folate), metabolic status (serum ferritin for iron, serum retinol for vitamin A, serum vitamin B_12_, and erythrocyte folate), and functional outcomes (anemia and neural tube birth defects), the effect of mass fortification is intertwined with the effects of other interventions. Williams et al. [4], in a separate paper in this supplement, described a process for conducting assessments for anemia and its causes. The same process also applies to fortification, in which practitioners can use nutrition surveillance data to design new or revise ongoing fortification programs by using information on clinical or functional changes measured frequently (yearly), and reserving biomarker data for measuring impact over longer periods (e.g., every 5 y). If these assessments are conducted before implementation of fortification, they can help reduce exposure to high levels of the micronutrient among population groups that are not targeted by the intervention.

When appropriately designed and implemented, food fortification supplements diets, and therefore prevents or reduces micronutrient inadequacies and associated deficiencies [149]. Although fortification complements the nutritional value of diets, practitioners may need to implement other interventions to correct micronutrient gaps in all population groups. In combination with other micronutrient-delivering interventions, food fortification should ensure that the intake of micronutrients is high enough to prevent inadequacies, without exceeding upper tolerable levels of intake. If implemented through centralized and reasonably developed factories and with reliable and efficient enforcement systems, food fortification might be the most cost-effective approach to deliver micronutrients to the general population in the near future.

In many countries, multiple food commodities can be fortified. The main consideration should be which to fortify to establish a food fortification platform with the lowest cost, to reach those who need it the most, and without risk of providing excessive amounts of micronutrients to others.

Many implementation research questions remain regarding current food-based fortification approaches, including the following:

• How can advances in food science lead to better food matrices for micronutrients in their vehicles?
• How do we integrate biofortified crops into the food value chain on a large scale?
• How do we mitigate the effects of climate change on food production and quality?
• How can we select ideal context-specific approaches to food fortification?
• What is the ideal combination of assessment biomarkers and bioindicators to estimate the impact of food fortification and biofortification on health and nutrition outcomes?
• What frequency of nutrition monitoring is required?
• How can we ensure that food manufacturers consistently fortify food per the country's standard?
• How do we design a program that supplies enough micronutrients to eliminate inadequacies without exceeding upper tolerable intakes in some population groups?
• How do we address gaps related to targeted fortification and its impact on anemia—recommended nutritional amounts of iron for children aged <2 y, the most bioavailable formulation, and most efficacious form of targeted fortification?
• How do we increase the percentage of total staple food supplies that are biofortified?
• How do we deliver fortified food products at the lowest costs to the consumer?

## Supplementation

Direct oral supplementation is another strategy to correct specific nutritional deficiencies linked to anemia. Although several micronutrients are needed to prevent and reduce anemia, the following sections cover aspects of iron supplementation because it is the micronutrient most difficult to deliver in sufficient amounts through other interventions.

### Iron supplementation

Oral iron supplementation is the first line treatment of iron deficiency and IDA in women [150]. Women of reproductive age in low-income countries are at high risk of both health problems, and WHO guidelines for weekly or daily population-based iron dosing schedules aim to prevent anemia in menstruating women in settings where anemia prevalence is ≥20% [151] or ≥40%, respectively (Table 2) [[152], [153], [154]]. On a global scale, anemia from chronic blood loss because of menstruation impacts a fifth of women, with the highest burden among those in low-income settings [[155], [156], [157]].

**TABLE 2:** WHO recommendations for prevention of iron deficiency anemia in menstruating women using weekly or daily oral iron supplementation

Recommendation	Dose (weekly)	Dose (daily)
Supplement	Iron: 60-mg elemental iron Folic acid: 2800 μg	30–60 mg elemental iron
Frequency	One supplement per week	One supplement daily
Duration	3 mo of supplementation followed by 3 mo of no supplementation, after which the provision of supplements should restart	Three consecutive months in a year
Settings	Populations where the prevalence of anemia among nonpregnant women of reproductive age is ≥20%	Populations where the prevalence of anemia in menstruating adult women and adolescent girls is ≥40%

60-mg elemental iron equals 300-mg FeSO_4_ heptahydrate, 180-mg ferrous fumarate or 500-mg ferrous gluconate. Modified from WHO [153,154].

Anemia, if caused by iron deficiency, usually responds rapidly to effective oral iron therapy and an Hb increase of ≥2 g/dL after 3 wk of therapy indicates adequate therapeutic response [158]. Iron preparations available on the market vary widely in dosage, formulation, cost, and bioavailability. Ferrous iron is preferred because of its high bioavailability (Table 3) [159]; however, the least expensive form, ferrous sulfate—and therefore the most commonly used—causes more gastrointestinal discomfort [160]. Ferric iron has low solubility at near-neutral or alkaline pH and must be reduced to ferrous iron before uptake by enterocytes [161]. Therefore, iron bioavailability from ferric iron preparations is typically 3–4 times lower than that of ferrous sulfate [119,162,163]. Compared with ferric iron, ferrous iron is generally more effective in replenishing Hb in patients with IDA [163]. For avoiding gastrointestinal problems, other ferrous compounds have been used, but they are more expensive as for example ferrous bisglycinate [[164], [165], [166]]. The bioavailability of ferrous bisglycinate (chelated form of iron) is high and they have been used in public health strategies to improve anemia [167,168].

**TABLE 3:** Iron content and relative bioavailability^1^ (RBV) of ferrous sulfate of commonly used oral iron preparations

Iron salt	Elemental iron content (% iron in formulation)	RBV to ferrous sulfate (%)
Ferrous sulfate	20	Reference
Ferrous sulfate (dried)	32.5	100
Ferrous fumarate	33	100
Ferrous gluconate	12	89
Amino acid chelates (e.g., ferrous biglycinate)	20	100
Carbonyl iron	99	5–20
Sugars (saccharides), usually ferric iron	Variable	Variable

1 Estimates of bioavailability of iron compounds are mainly derived from their comparisons at low doses as food fortificants rather than as supplements. Bioavailability data of iron fortificants may not be entirely applicable to oral iron preparations, but it remains the best evidence of relative bioavailability of oral supplements.

A recent meta-analysis of 20 trials showed an increased incidence of gastrointestinal side effects compared with placebo when oral ferrous sulfate (dosage ranging from 20 to 400 mg iron/d) was given (odds ratio: 2.32, 95% CI: 1.74, 3.08, *P* < 0.0001) [169]. Equal doses of iron as ferrous sulfate, ferrous fumarate, and ferrous gluconate in healthy adults resulted in no significant differences in side effects [170]. The most common adverse effects are epigastric pain, nausea, and constipation; these reduce compliance with therapy in 30%–70% of cases [169]. Oral doses ≤50 mg iron/d generally cause less severe side effects than higher doses [171].

The patient’s diet can also affect the efficacy of supplementation, as many foods and drinks contain inhibitors of iron absorption. Fractional iron bioavailability from supplements varies from 2 to 13% when consumed with food compared with 5%–28% when fasting [172]. Whole grains and pulses are rich in phytic acid, a strong inhibitor of iron absorption at even low levels (a molar ratio of phytate to iron of >1). Coffee, black tea, herbal teas, red wine, and hot chocolate contain polyphenols, which also impair iron bioavailability [173]. Consuming a food or drink rich in ascorbic acid with oral iron can sharply increase bioavailability; especially at a molar ratio ≥2:1 (e.g., 60-mg ascorbic acid:10-mg iron) [173]. As discussed by Brittenham et al. [3] elsewhere in this supplement, the primary regulator of body iron homeostasis is the hepatic peptide, hepcidin. We highlight relevant aspects of hepcidin biology to the use and effectiveness of iron supplements (Supplemental File, page 8). To summarize, oral doses of ferrous sulfate ≥60 mg iron in nonanemic women with iron deficiency and ≥100 mg in women with IDA triggers an increase in circulating hepcidin that persists for 24 h after the dose, but subsides within 48 h. To maximize fractional iron absorption, give oral doses on alternate days: alternate day dosing increases fractional iron absorption by 34%–50% compared with the same dose given on consecutive days. There is a circadian increase in circulating hepcidin during the day. Because this is augmented by a morning iron dose, iron doses should not be given in the afternoon or evening after a morning dose.

Pregnant women require additional iron and folic acid to meet their own needs as well as those of the fetus. WHO recommends daily oral iron and folic acid supplementation with 30–60 mg elemental iron and 400 μg (0.4 mg) folic acid to prevent maternal anemia, low-birth weight, and preterm birth [153]. Although the quality of the evidence remains moderately low, based on the quality of included studies in the systematic review done in 2018, oral iron supplements (60-mg elemental iron and 400 -μg folic acid) taken intermittently 3–5 times/wk, reduce IDA [174]. The WHO also suggests the use of “intermittent iron and folic acid supplements by nonanemic women as a recommended alternative to prevent anemia and improve gestational outcomes in areas where the prevalence of anemia among pregnant women is lower than 20%” [151]. The weekly dose is suggested where the consumers report increased frequency of side effects, as the participants report better adherence to the weekly dose. The weekly dose is used in the prevention of anemia. The widespread use of school- or community-based weekly IFA supplementation has also been shown to reduce adolescent anemia [175].

As discussed above, supplemental iron may increase the risk of malaria and other infections [176]. In a large intervention trial in Tanzania, there was a higher incidence of severe adverse events (hospitalizations from malaria and other infections and deaths) in children provided supplements with iron and folic acid compared with placebo [177]. WHO and UNICEF urge caution with regard to iron supplementation in areas with high infection burden [178]. Iron supplementation in these areas should be accompanied by strategies to prevent and treat malaria [179]. Data from a recent double-blind, placebo-controlled trial in mostly iron-sufficient infants in Bangladesh of 3 mo of daily supplementation with iron syrup or multiple MNPs reported reduced rates of anemia but no improvement in cognitive development [180,181]. The investigators conducted economic analysis and simulation studies with data from the trial that support the assertion that iron or MNP supplementation for anemia may only be cost effective in regions where the prevalence of iron deficiency is high, adherence to supplements is high and infections, such as malaria, do not play a major causative role [182,183]. As outlined by Brittenham et al. [3], consider the risk–benefit in the context of underlying infection [184]. We outline the research needs regarding the safe and efficacious use of iron supplements (Supplemental File, page 8).

### Multiple micronutrient supplements and other micronutrients of interest

Current evidence suggests that giving multiple micronutrient supplements (MMS) containing 13–15 micronutrients including iron to pregnant women may reduce the risk of low-birth weight and of small for gestational age, compared with iron and folic acid supplementation alone. However, there is also evidence of risk in some subgroups (neonatal mortality), as well as important gaps, in the evidence. At this time, WHO does not recommend multiple micronutrient supplementation for pregnant women but allows its use under the context of rigorous research [185]. As per the WHO, rigorous research in the context of their guideline includes the following: “*1*) Controlled clinical trials in which early pregnancy ultrasound is used to establish gestational age with certainty, with assessment of critical maternal and perinatal outcomes, and follow-up of infants sustained into childhood; and *2*) where programs of MMS are being considered, implementation research to establish the impact of switching from iron and folic acid supplements to MMS, including evaluation of acceptability, feasibility, sustainability, equity and cost-effectiveness” [186].

### MNPs, the gut microbiome, and diarrhea in infants

MNPs containing iron along with multiple vitamins and minerals for home food fortification of infant complementary foods has been recommended for infants and young children between 6 and 23 mo and is widely used as a promising strategy to combat micronutrient deficiencies. Using iron-containing MNPs along with complementary foods effectively reduces the risk for IDA in infants [187]. Iron syrup and MNPs appear to have similar effects in reducing the prevalence of anemia and ID [180].

However, this delivery strategy of iron intervention is not without risk. The 10-mg iron dose [188] in multiple MNP sachet of 1 g is high—comparable with an mg of iron per kg body weight to supplemental doses (2 mg/kg) typically given to older children. Moreover, the absorption of MNP iron is low, usually <10% [189]; most of the iron passes into the infant colon unabsorbed. In controlled studies, MNPs modestly increase the risk for diarrhea in infants; in some cases, the diarrhea is severe and may require hospitalization [179,190]. Potential impact on the gut microbiome from the provision of iron-containing MNPs to infants can decrease the number of beneficial “barrier” commensal gut bacteria (e.g., bifidobacteria), increase opportunistic pathogens (e.g., *Escherichia coli*), and induce gut inflammation [191,192]. To minimize these detrimental effects, provide the lowest effective dose and maximize fractional iron absorption. Prebiotic galacto-oligosaccharides may prove useful in iron formulations in low- and middle-income countries because they enhance the absorption of iron [193] and may mitigate adverse effects of unabsorbed iron on the infant gut [194].

### IV iron therapy

IV iron warrants attention here as a method of delivering iron directly to individual patients under conditions that oral iron is not possible to administrate. IV iron therapy is usually reserved for patients with IDA who are unresponsive or intolerant to oral iron, with chronic kidney disease [195], or when rapid correction of the iron deficit is required (e.g., preoperatively) [196]. IV iron formulations are nanoparticles composed of carbohydrate ferric oxyhydroxides. Available formulations include iron dextran, iron sucrose, ferric carboxymaltose, iron isomaltoside, ferrumoxytol, and ferric gluconate [196]. Titrated doses can correct the total body iron deficit with single or repeated administration. Several formulations, i.e., ferric carboxymaltose and iron isomaltoside, enable very high doses (∼1000 mg or more) to be safely given in short infusion times. In prospective trials, the risk of moderate to severe infusion reactions was similar among these preparations and affected <1% of patients [197].

These results, importantly, highlight the role of the ecology of anemia in selecting the appropriate interventions—the adoption of universal implementation of interventions has to be tempered with an understanding of biology and assessment of the etiology of anemia.

### Other anemia-relevant micronutrient supplementation

In addition to supplying iron, vitamin A, folate, and vitamin B_12_ are micronutrients of interest when considering supplementation as an intervention to address anemia. Although zinc and iron share common transport mechanisms in the gastrointestinal tract, the role of zinc deficiency in causation of anemia remains a gap in the knowledge, however, the coexistence of zinc and iron deficiency can be explained through biological mechanisms [198] and is also seen in population studies [199]. Foy and Mbaya [200] also identify riboflavin as a vitamin whose severe deficiency leads to anemia. In metabolic studies, severe prolonged riboflavin deficiency produces anemia. The prevalence of riboflavin deficiency in the general population is unknown but, in general, not expected to be a public health problem. A narrative literature review evaluating these and other micronutrients concluded that although provision of multivitamins across age groups (preschool and school children including adolescents) reported increases in Hb concentration and reductions in anemia; these effects were indistinguishable from those seen with iron alone [88]. Although neonatal vitamin A supplementation reportedly did not impact the risk of anemia [201], vitamin A supplementation during pregnancy was associated with reduced risk [202]. Vitamin B_12_ supplementation can increase Hb concentration in women and children who receive a supplement compared with those who do not [203]. Clinical vitamin B_12_ deficiency with classic hematologic and neurologic manifestations is relatively uncommon but can manifest in up to a quarter of the population as a subclinical deficiency in studies where it is measured [204]. Vitamin B_12_ supplementation would work for those who are deficient because of insufficient intake and suffer from malabsorption because of gastric atrophy. The recommended daily allowance is 2.4 mg. If the malabsorption is caused by pernicious anemia, administer high doses of vitamin B_12_ in the form of a supplement to meet the body’s needs in spite of the inefficient process of passive absorption of the vitamin. There is scant evidence to support supplementation of vitamin A, folate, and vitamin B_12_ for the purpose of reducing anemia. A well-designed fortification program can meet the needs for these micronutrients—at doses that are practical and only in individuals who lack it because of insufficient intake, not malabsorption.

### Summary for nutritional-specific interventions

Research has demonstrated the benefits of population-mass and targeted food fortification, oral and IV iron supplementation, folate fortification and supplementation, and selective micronutrient supplementation as effective interventions to address the issue of anemia. Food-based interventions, such as addition of animal source foods to diet and various food-processing techniques have potential to make an impact, if issues related to scalability can be addressed.

In conclusion, we have critically reviewed the relative strengths and weaknesses of the range of potential nutritional/dietary or nonnutritional interventions to address anemia. Table 4 provides a summary of key contextual considerations as they pertain to specific intervention options to reduce anemia in populations from a public health perspective. The table lists 5 key characteristics of interventions before considering implementing them, whether singly or jointly. This includes efficacy of an intervention, its effectiveness, the potential to scale the interventions over a population or target it to a specific group, interactions between interventions, and potential social and behavior change considerations. We recognize that program managers balance available resources with competing priorities, and Table 4 serves as an aide memoire for translating biology and assessment information into program action. Our goal has been to refocus our perspectives of anemia to view it as a complex ecology. We review assessment and intervention approaches to support context-specific solutions for addressing anemia in any of its forms clinically and from a public health perspective. We hope that this more integrated approach will support global efforts to positively change the course of this public health challenge.

**TABLE 4:** Approaches to implementation of interventions to address anemia

Intervention	Efficacy	Effectiveness	Scalability	Interactions	Social and behavior change considerations
Nondietary interventions					
Malaria preventive chemoprophylaxis	Proven	Proven	Already scaled up	In areas with poor coverage of malaria prevention and treatment, iron supplementation may increase morbidity and mortality	Usage of insecticide nets, fear of side effects from chemoprophylaxis, and lack of adherence when moving from treatment to prevention
Antihelminth treatment	Proven	Effectiveness confounded by interactions with malnutrition, poverty, low educational levels, and socioeconomic status. Proven at the community level in combination with water, sanitation, and hygiene and iron and vitamin A supplementation programs	Programs are scaled up	Programmatically, used in conjunction with prenatal iron supplementation (in sub-Saharan Africa and Latin America) and vitamin A and/or folic acid supplementation (in Asia)	Need to address provider and caregiver behaviors
Treatment of anemia of inflammation caused by tuberculosis and HIV	Proven—when addressing underlying cause	Proven—anemia improves with the treatment of tuberculosis and HIV	Programs are scaled up	Additional supplementation with iron, folic acid, and vitamin B_12_	Related to tuberculosis and HIV programs
Management of acute blood loss	Proven—when addressing underlying cause	Implementation challenges in health facilities	Even with facility resources, the uptake is low	Blood transfusions	Relates to provider training and preparation
Management of chronic blood loss	Evidence quality is low	Implementation challenges	Even with facility resources, the uptake is low	Iron supplementation	Relates to provider training and preparation
Delayed cord clamping	Proven	Proven and used widely	Scaled up	Alongside other interventions for a positive pregnancy experience	Need to address provider behaviors, training, and preparation
Management of inherited blood disorders	Supportive and palliative care	More research needed	More research needed	Interactions with iron supplementation and malaria programs	
Dietary enhancement and diversification					
Addition of animal sources of iron to foods and improving dietary diversity to enhance iron intake and absorption	Mixed effects, not conclusive Systematic review shows benefit of dietary diversity and animal source food consumption More evidence needed on dose–response relationship Larger randomized trials needed	Not yet	More research needed	Food–food /nutrient–nutrient interactions Infections Iron status Public health interventions such as antenatal and postnatal care, and supplementation with other nutrients (vitamin A, folic acid, ascorbic acid, etc.)	
Food processing to improve iron bioavailability	Limited information from small studies Mixed effects Greatest potential shown in dehulling, phytase enzyme, and extrusion cooking	Not yet	Limited scalability Opportunity cost because of labor intensive traditional processing techniques More research needed	Processing method, time, temperature, pressure Food matrix: dietary fiber, antinutrients Nutrient–nutrient interactions (vitamin A, ascorbic acid, B_6_, B_12_, vitamin E, riboflavin, folic acid; minerals such as calcium, copper, zinc, selenium, etc.) Infections Iron status	
Food-based fortification approaches					
Mass food fortification	Proven	Proven	Scaled up	In conjunction with other context- and need-based interventions that supply micronutrients	Effective mass fortification builds on, rather than changes, the normal eating habits of the population; social marketing is therefore often not necessary
Targeted food fortification	Proven	Proven in specific contexts	Not used widely (mainly used in select population groups)	Selective use of this intervention, based on context	
Biofortification	Proven	Increasing	Integration into agricultural value chains and food systems	Potential synergy with dietary diversification approaches	Needed for vitamin A biofortification, because beta-carotene content turns crops yellow or orange
Supplementation					
Iron supplementation (oral)	Proven	Proven	Already scaled up	May have reduced impact in areas with high amounts of inflammation/infection; may increase morbidity and mortality in areas with high malaria burden and poor health infrastructure	
Iron supplementation (IV therapy)	Proven in the clinical setting	Not yet used on a population basis	Not scaled up	Safety not yet widely evaluated in low-resource settings	
Micronutrient powders	Proven	Local sustainability is still questionable in many settings	Already scaled up	May cause dysbiosis, gut inflammation, and diarrhea in low-hygiene areas	MNPs are not likely cost-effective interventions in terms of costs per disability-adjusted life years, especially where anemia is less prevalent or the severity of anemia is not high
Other micronutrients supplementation	Mostly unproven; limited evidence for folic acid and vitamin A	Unproven	Folic acid scaled up in some countries for prophylaxis of neglected tropical diseases	Uncertain	

## Author contributions

The authors’ responsibilities were as follows – all authors: jointly designed the review and wrote the paper; DM: has primary responsibility for final content; and all authors: read and approved the final manuscript.

## Conflict of interest

The authors report no conflicts of interest.

## Funding

This article is published as part of a supplement sponsored by JSI Research & Training Institute, Inc. This manuscript was developed by the USAID Advancing Nutrition Anemia Task Force (ATF), which is supported by the U.S. Agency for International Development. The review manuscript was prepared under the terms of contract 7200AA18C00070 awarded to JSI Research & Training Institute, Inc. (JSI). USAID staff participated in the ATF meetings and working groups in their capacity as anemia experts and served as coauthors of the review papers.

## Disclaimers

The contents of this manuscript are those of the authors and do not necessarily represent the official position of the United States Agency for International Development, the National Institutes of Health, or the U.S. government.
