# Supplementary material for: Approaches to Address the Anemia Challenge
Source: J Nutr. 2023 Sep 14;153(Suppl 1):S42–59. doi: 10.1016/j.tjnut.2023.07.017 (PMC10797550; doi:10.1016/j.tjnut.2023.07.017)
Supplement: Multimedia component 1 [file mmc1.docx]

**Approaches to Address the Anemia Challenge**

Cornelia U Loechl,^1^ Ananya Datta Mitra,^2^ Lindy Fenlason,^3^ Ralph Green,^2^ Laura Hackl,^4^ Laura Itzkowitz,^3^ Marion Koso-Thomas,^5^ Denish Moorthy,^4^ Victor Ochieng Owino,^1^ Helena Pachón,^6^ Nicole Stoffel,^7^ Michael Zimmerman,^7^ Daniel J Raiten^5^

^1^ Division of Human Health, International Atomic Energy Agency, Vienna, Austria

^2^ Department of Pathology and Laboratory Medicine, University of California, Davis, USA

^3^ Bureau for Global Health, USAID, Washington, DC

^4^ USAID Advancing Nutrition, Arlington, VA

^5^ Eunice Kennedy Shriver National Institute of Child Health and Development, National Institutes of Health, USA

^6^ Food Fortification Initiative, Atlanta, USA

^7^ Human Nutrition Laboratory, Swiss Federal Institute of Technology (ETH), Switzerland

**Corresponding Author:** Denish Moorthy, 2733 Crystal Dr 4th floor, Arlington, VA 22202; [denish_moorthy@jsi.com](mailto:denish_moorthy@jsi.com)

**Supplemental Table 1: Hemoglobin levels to diagnose anemia in children**

| **Child Age group** | **Hemoglobin cutoff for Anemia in g/dL** |
| --- | --- |
| Children 6 - 59 months of age | < 11 |
| Children 5 - 11 years of age | <11.5 |
| Children 12 - 14 years of age | <12 |

**Research Questions and Future Directions for Non-Nutritional Interventions for Anemia.**

- Develop empiric guidelines for duration of delayed cord clamping by gestational age and relevant maternal risk factors.
  - Determine true frequency of delayed cord clamping and assess cost benefit to scale-up practice.
  - Develop algorithms of delayed cord clamping for birth attendants.
  - Understand drug interactions and safety of mass deworming programs in contexts with endemic malaria and HIV.
  - Identify factors impacting on compliance of large-scale deworming programs.
- Quantify co-infections that cause anemia in pregnancy and childhood and match targeted population level programs with the prevalence.
- Identify and develop strategies to address new and emerging parasitic infections from fish among affluent pregnant populations.
- Improve understanding and implications of urinary, vaginal, intestinal, skin microbiome in pregnant and lactating mothers for susceptibility to and treatment of anemia associated infectious disease.
- Improve understanding of factors influencing changes in Plasmodium immunity as drug resistance increases and maternal response changes particularly in the context of relevant anemia-related conditions.
  - Improve, develop, and enhance deployment of tools for sero-surveillance of malaria in pregnancy.
  - Explore the potential benefit of malaria vaccination on anemia prevalence among these populations
- Determine the feasibility and scientific merit for a broad approach to reduction of inflammation as an intervention for treatment of anemia.

**Implications of Climate Change for Choice of Nutritional Intervention— Addressing Nutritional Iron Deficiency and Malaria .** We illustrate the challenges raised by the impact of climate change on the decision to adopt a particular intervention, in this example it is the addressing iron deficiency in the context of malaria.

1. Option 1: Improve dietary diversity

Challenges-

- - Climate change’s impact on resources (land/water/human)
  - Can we produce enough food of high enough quality including iron content in a high CO_2_ environment?
  - Can we increase the intake of indigenous/culturally acceptable and affordable (land/marine) animal source foods without further exacerbating the impact on global greenhouse gas emissions (GHGE)/climate?

1. Option 2: Food fortification/biofortification, Increase of commercially prepared foods

Challenges-

- Resource/capacity for local fortification efforts
- Implications of GHGE/CO2 on expectations of improved nutrient levels in biofortified foods
- Potential of the “nutrition transition” in changing traditional diets and potential implications for unintended consequences (e.g., increased NCDs).

1. Option: Supplementation

Challenges-

- Safety and effectiveness of iron supplements in the context of malaria and/or other endemic infections, which show increased transmission in countries that are impacted by climate change
- Is the standard public health approach to supplementation (i.e., one-size-fits-all) safe in the context of a complex health scenario that includes single or more often multiple co-morbidities superimposed on food/nutrition insecurity?

*In Vitro Studies to Reduce Anti-Nutrients*

*Fermentation, Germination*

A body of evidence exists from in vitro studies of the potential benefits of these types of food manipulations. In an extensive review of in vitro studies, Mensah and Tomkins argued fermentation and germination increase the bioavailability of iron from cereals and whole grains with a typical high phytic acid: iron molar ratio (1). Estimates suggest that phytate content can be reduced by up to 90 percent by fermentation, 50–64 percent by germination, and 47–98 percent by soaking, depending on the staple food and methods used (2).

Hemalatha and colleagues found that both germination and fermentation increased iron bioavailability and reduced tannin and phytic acid concentrations in various cereal-pulse combinations (3). Mihafu and co-workers found that while a 72-hour germination process significantly increased iron content and reduced phytic acid concentration in maize, it resulted in increased concentration of polyphenols (4). Trained health workers in Kenya included germinated amaranth in a complementary food blend, but this did not result in any significant improvement compared to fortified corn-soy blend in either hemoglobin or iron stores among infants after nine months of intervention (5). Scheers et al. reported that lactic-fermented vegetables served with both a high-phytate and low-phytate meal increased the bioavailability of iron but not zinc among 17 adults aged 21–54 years (6). Asres and colleagues also observed decreased phytic acid and tannin content in teff and wheat cereals, but the effect was at its maximum after 72 hours of spontaneous fermentation (7). However, both studies observed wide variation in iron content of fermented products and they are based on in vitro iron bioavailability assessment. The feasibility of scale-up of fermentation is also limited since an energy-consuming drying step is a prerequisite for safe storage, and fermentation and subsequent drying can be critical points for pathogenic contamination.

Feitosa and co-workers showed that soaking common beans before pressure cooking them without discarding the soaking water may maximize iron and zinc bioavailability (8). This is most likely due to hydrolysis of phytic acid and polyphenols under high temperature and pressure and the retention of iron in cooking water (9). Perlas and Gibson found that soaking rice and mung beans for up to 12 hours can reduce phytate content by up to 98 percent (10). Temple and colleagues showed that soaking maize and decanting the water improved both phytate: iron and phytate: zinc molar ratios in a complementary food for infants in Malawi (11).

*Dephytinization, Abrasive Decrotification, and Dehulling*

Gibson and colleagues stated that dephytinization of cereals could enhance iron, zinc, and calcium absorption (12). Some traditional processing methods like abrasive decortication, the removal of outer seed layers, target phytate reduction but lead to significant loss of iron, zinc, and lipids (13) with only partial reduction in phytate content (14). Dehulling to remove parts with highest phytic acid concentration can help increase iron bioavailability 12-fold (15), especially in cereals where the phytic acid is mainly located in the seed coat (16).

*Native or Exogenous Phytase Enzyme*

The benefit of fungal phytase enzyme has been described (17–19), but the hope of wide application of this approach has not been realized, as approval for commercial production has not been successful. Hurrell and colleagues found that the addition of exogenous phytase improved iron bioavailability in roller dried complementary foods composed of rice, wheat, maize, oat, sorghum, and a wheat-soy blend (20). Similarly, Herter-Aeberli and colleagues demonstrated that activation of native phytase or the addition of phytase from *Aspergillus niger* to a FeSO_4_ fortified injera increased fractional iron bioavailability (21). Replacement of injera with 10 percent whole wheat flour (which contains native phytase) tripled fractional iron bioavailability from the fortified injera (21).

*Extrusion Cooking*

Lastly, extrusion cooking is thought to contribute indirectly to increased mineral bioavailability through a hydrolytic reducing effect on phytic acid, polyphenols, and dietary fiber (22), however the evidence on iron bioavailability in humans is limited. The temperature-pressure combination is a major factor, with some studies showing that cold extrusion has greater benefit on iron bioavailability since it doesn’t drastically modify starch crystalline structure (22–24). The blend containing germinated amaranth, both fortified with multiple micronutrients and not, and the fortified corn-soy blend, had comparable effects on fat free mass and attained length. Scheers et al. reported that lactic-fermented vegetables served with both a high-phytate and low-phytate meal increased the bioavailability of iron but not zinc among 17 adults aged 21–54 years (6). These two studies illustrate how germination can address iron intake in various age groups, however, their comparison is limited by the differences in biological ecologies of infants versus adults. Despite the potential of fermentation, the feasibility of scale-up is limited; an energy-consuming drying step is a prerequisite for safe storage, and most often fermentation and subsequent drying can be critical points for pathogenic contamination.

**Relevant Aspects of Hepcidin Homeostasis and Iron Supplementation**

- During IDA, circulating hepcidin levels fall and intestinal iron absorption increases.
- Plasma hepcidin not only responds to changes in body iron stores, its synthesis is also stimulated by high doses of oral iron.
- Oral doses of ferrous sulfate ≥60 mg in non-anemic women with iron deficiency and ≥100 mg in women with IDA trigger an increase in circulating hepcidin that persists for 24 hours after the dose, but subsides within 48 hours.
- To maximize fractional iron absorption, give oral doses on alternate days: alternate day dosing increases fractional iron absorption by 34–50% compared to the same dose given on consecutive days.
- There is a circadian increase in circulating hepcidin during the day. Since this is augmented by a morning iron dose, iron doses should not be given in the afternoon or evening after a morning dose.

**Future Directions and Key Research Questions for Supplementation**

- Increase understanding of the role of iron supplementation in various infectious diseases, including malaria, HIV disease, tuberculosis, pneumonia, diarrheal disease, and COVID-19.
- Develop safer formulations of oral iron supplements and iron containing MNPs that reduce the potential adverse effects of iron on the gut.
- Clarify the mechanisms of intestinal heme absorption as a potential well-absorbed form of oral iron supplementation.
- Improve iron status markers and assessment to better evaluate impact of iron supplementation and differentiate between functional and absolute iron deficiency.
- Develop optimized protocols for iron supplementation to reduce side effects and increase compliance based on iron and hepcidin physiology.
- Provide clearer indications for use of high-dose IV iron.
- Improve understanding of the benefits and risks of iron supplementation during pregnancy and birth outcomes.
- Assess the impact of iron supplementation in pregnancy and children on neurologic and cognitive development.
- Contribute to the understanding of the effect of Hb genotypes on risk of iron deficiency and response to iron supplementation.
- Increase understanding of the risks and benefits of iron supplementation in noncommunicable diseases (e.g., congestive heart failure, cancer).

**Supplemental Table 2. Number of Countries with Selected Nutrients Included in Standards for Mandatory Fortification, and the Levels of Those Nutrients that Must Be Added through Fortification in parts per million (ppm)**

| **Crop** | **Mandatory Legislation (# of Countries)** | **Iron** | **Vitamin A** | **Folic Acid** | **Vitamin B_12_** | **Zinc** |
| --- | --- | --- | --- | --- | --- | --- |
| Maize flour | 16 | 16 countries: 10–65 ppm | 9: 0.5–2.7 | 15: 1–2.6 | 9: 0.003–0.02 | 11: 15–50 |
| Milk | 14 | 1: not specified^1^ | 11: not specified^1^ | 1: not specified^1^ | 0 | 0 |
| Oil | 26 | 0 | 22: 6–42 | 0 | 0 | 0 |
| Rice | 7 | 6: 24–75 | 0 | 5: 1–2.31 | 3: 0.01 | 4: 7–45 |
| Salt | 129 | 2: 10–980 | 0 | 0 | 0 | 0 |
| Wheat flour | 83 | 77: 10–120 | 15: 1–10 | 62: 0.4–5.12 | 16: 0.01–0.04 | 25: 15–102 |

Ppm, parts per million; ^1^ The nutrient levels are not specified Source: (25,26).

**References:**

1. Mensah P, Tomkins A. Household-level technologies to improve the availability and preparation of adequate and safe complementary foods. Food Nutr Bull 2003;24:104–25.

2. Dewey KG. Increasing iron intake of children through complementary foods. Food Nutr Bull 2007;28.

3. Hemalatha S, Platel K, Srinivasan K. Influence of germination and fermentation on bioaccessibility of zinc and iron from food grains. Eur J Clin Nutr 2007;61:342–8.

4. Mihafu F, Laswai HS, Gichuhi P, Mwanyika S. Influence of Soaking and Germination on the Iron, Phytate and Phenolic Contents of Maize Used for Complementary Feeding in Rural Tanzania. Int J Nutr Food Sci 2017;6:111.

5. Konyole SO, Omollo SA, Kinyuru JN, Skau JKH, Owuor BO, Estambale BB, Filteau SM, Michaelsen KF, Friis H, Roos N, et al. Effect of locally produced complementary foods on fat‐free mass, linear growth, and iron status among Kenyan infants: A randomized controlled trial. Matern Child Nutr [Internet] 2019 [cited 2021 Apr 21];15. Available from: https://onlinelibrary.wiley.com/doi/abs/10.1111/mcn.12836

6. Scheers N, Rossander-Hulthen L, Torsdottir I, Sandberg A-S. Increased iron bioavailability from lactic-fermented vegetables is likely an effect of promoting the formation of ferric iron (Fe3+). Eur J Nutr 2016;55:373–82.

7. Asres DT, Nana A, Nega G. Complementary feeding and effect of spontaneous fermentation on anti-nutritional factors of selected cereal-based complementary foods. BMC Pediatr 2018;18:394.

8. Feitosa S, Greiner R, Meinhardt A-K, Müller A, Almeida D, Posten C. Effect of Traditional Household Processes on Iron, Zinc and Copper Bioaccessibility in Black Bean (Phaseolus vulgaris L.). Foods 2018;7:123.

9. Deol JK, Bains K. Effect of household cooking methods on nutritional and anti nutritional factors in green cowpea (Vigna unguiculata) pods. J Food Sci Technol 2010;47:579–81.

10. Perlas LA, Gibson RS. Use of soaking to enhance the bioavailability of iron and zinc from rice-based complementary foods used in the Philippines. J Sci Food Agric 2002;82:1115–21.

11. Temple L, Gibson RS, Hotz C. Use of Soaking and Enrichment for Improving the Content and Bioavailability of Calcium, Iron, and Zinc in Complementary Foods and Diets of Rural Malawian Weanlings. J Food Sci 2002;67:1926–32.

12. Gibson RS, Perlas L, Hotz C. Improving the bioavailability of nutrients in plant foods at the household level. Proc Nutr Soc 2006;65:160–8.

13. Hama, Icard-Vernière C, Guyot, D. Brehima, and Claire Mouquet. J-P, Brehima D, Mouquet C. Iron and zinc bioavailability during abrasive decortication of non-gmo biofortified and traditional pearl millet. Ann Nutr Metab 2011;58.

14. Hama, Icard-Vernière C, Guyot J-P, Rochette I, Diawara B, Mouquet-Rivier C. Potential of non-GMO biofortified pearl millet (Pennisetum glaucum) for increasing iron and zinc content and their estimated bioavailability during abrasive decortication: Iron and zinc in biofortified millet. Int J Food Sci Technol 2012;47:1660–8.

15. Hurrell RF. Phytic Acid Degradation as a Means of Improving Iron Absorption. Int J Vitam Nutr Res 2004;74:445–52.

16. Lestienne I, Mouquet-Rivier C, Icard-Verniere C, Rochette I, Treche S. The effects of soaking of whole, dehulled and ground millet and soybean seeds on phytate degradation and Phy/Fe and Phy/Zn molar ratios. Int J Food Sci Technol 2005;40:391–9.

17. Cercamondi CI, Egli IM, Mitchikpe E, Tossou F, Zeder C, Hounhouigan JD, Hurrell RF. Total iron absorption by young women from iron-biofortified pearl millet composite meals is double that from regular millet meals but less than that from post-harvest iron-fortified millet meals. J Nutr 2013;143:1376–82.

18. Nielsen AV, Meyer AS. Phytase-mediated mineral solubilization from cereals under *in vitro* gastric conditions: Phytase-mediated mineral release. J Sci Food Agric 2016;96:3755–61.

19. Troesch B, Jing H, Laillou A, Fowler A. Absorption Studies Show that Phytase from *Aspergillus niger* Significantly Increases Iron and Zinc Bioavailability from Phytate-Rich Foods. Food Nutr Bull 2013;34:S90–101.

20. Hurrell. Degradation of phytic acid in cereal porridges improves iron absorption by human subjects. Am J Clin Nutr 2003;77:1213–9.

21. Herter-Aeberli I, Fischer MM, Egli IM, Zeder C, Zimmermann MB, Hurrell RF. Addition of Whole Wheat Flour During Injera Fermentation Degrades Phytic Acid and Triples Iron Absorption from Fortified Tef in Young Women. J Nutr 2020;150:2666–72.

22. Singh S, Gamlath S, Wakeling L. Nutritional aspects of food extrusion: a review. Int J Food Sci Technol 2007;42:916–29.

23. Hackl L, Speich C, Zeder C, Sánchez-Ferrer A, Adelmann H, de Pee S, Tay F, Zimmermann MB, Moretti D. Cold Extrusion but Not Coating Affects Iron Bioavailability from Fortified Rice in Young Women and Is Associated with Modifications in Starch Microstructure and Mineral Retention during Cooking. J Nutr 2017;147:2319–25.

24. Colonna S, Monticelli O, Gomez J, Saracco G, Fina A. Morphology and properties evolution upon ring-opening polymerization during extrusion of cyclic butylene terephthalate and graphene-related-materials into thermally conductive nanocomposites. Eur Polym J 2017;89:57–66.

25. López de Romaña D, Verona S, Vivanco OA, Gross R. Protective effect of multimicronutrient supplementation against anemia among children, women, and adolescent girls in lower-income areas of Chiclayo, Peru. Food Nutr Bull 2006;27:S143-150.

26. Global Fortification Data Exchange. Interactive map: nutrient levels in fortification standards (mid-range or average) [Internet]. 2020 [cited 2021 Mar 1]. Available from: https://fortificationdata.org/map-nutrient-levels-in-fortification-standards/
